# Near-Infrared Fluorescence Imaging in General Surgery: Applications in Vascularization, Tumor Margin Detection, and Biliary Anatomy

**DOI:** 10.7759/cureus.92194

**Published:** 2025-09-13

**Authors:** Mohamed Abosheisha, Elmoatazbellah Nasr, Mohamed Ali, Rezuana Tamanna, Samir Bin Halim, Muhammad Rakib Hasan, Momen Abdelglil, Ahmed Swealem, Mohamed Ismaiel

**Affiliations:** 1 General Surgery, Wirral University Teaching Hospital NHS Foundation Trust, Wirral, GBR; 2 General Surgery, Calderdale and Huddersfield NHS Foundation Trust, Huddersfield, GBR; 3 Surgery, Hywel Dda University Health Board, Carmarthen, GBR; 4 General Surgery, Craigavon Area Hospital, Northern Ireland Medical and Dental Training Agency, Belfast, GBR; 5 General Surgery, Watford General Hospital, Watford, GBR; 6 Urology, Watford General Hospital, West Hertfordshire Teaching Hospitals NHS Trust, Watford, GBR; 7 Pediatric Surgery, Mansoura University Children's Hospital, Mansoura, EGY; 8 Orthopedics, Southmead Hospital, North Bristol NHS Foundation Trust, Bristol, GBR; 9 General Surgery, University Hospital Limerick, Limerick, IRL

**Keywords:** biliary anatomy, cancer surgery, cholangiography, fluorescent contrast agents, near-infrared fluorescence, nirf, nir-i and nir-ii fluorophores, robotic surgery, tumor margin detection, vascularization

## Abstract

Near-infrared fluorescence (NIRF) imaging has emerged as a transformative intraoperative tool in general surgery, helping in vascular surgery interventions, tumor margin detection in different specialties, and biliary anatomy with high sensitivity and safety. Indocyanine green remains the gold-standard fluorescent agent, widely applied in angiography, tumor margin detection, and fluorescence cholangiography, consistently improving surgical safety and efficiency with exceedingly low complication rates. Recent advances and trends include activatable and targeted probes, near-infrared II fluorophores, and nanoparticle-based agents, which expand specificity, depth of imaging, and multiplexing capacity. Integration with robotic systems in general surgery practice and artificial intelligence may further strengthen the precision of fluorescence-guided surgery, providing automated interpretation and workflow integration. Clinical outcomes demonstrate reduced operative times, improved anatomical visualization during operations, and enhanced tumor resection accuracy, while emerging technologies promise broader applications in complex oncologic and hepatopancreatobiliary surgery. Taken together, NIRF imaging represents a rapidly evolving field with the potential to redefine intraoperative guidance and precision in general surgery.

## Introduction and background

Near-infrared fluorescence (NIRF) imaging is an advanced optical technique that utilizes light within the wavelength range of 700-900 nm, allowing it to effectively penetrate both blood and biological tissues [1]. By combining this specific light spectrum with specialized high-resolution cameras and the administration of fluorescent contrast agents, surgeons are able to visualize structures that would otherwise remain concealed to the naked eye. This approach provides enhanced intraoperative guidance by clearly highlighting intricate anatomical details and outlining fine structural variations, thereby improving surgical precision and safety [1,2]. NIRF imaging has found wide clinical application across multiple surgical disciplines. It is most commonly employed for assessing tissue perfusion in vascular procedures, where it provides real-time information about the adequacy of blood flow [3]. In addition, it plays a valuable role in lymphatic surgery through lymph node mapping, and it is increasingly utilized in oncologic operations for accurate tumor localization and margin assessment [3,4].

Beyond these uses, NIRF is used in other fields such as general surgery, oncologic surgery, and minimally invasive approaches by enabling the clear identification of vital anatomical structures that are often difficult to visualize with conventional techniques, thereby enhancing both safety and surgical outcomes [3-8].

Indocyanine green (ICG) is the main fluorescent dye used for biliary system imaging. After intravenous injection, it binds mostly to albumin and is cleared through the liver. When excited by near-infrared (NIR) light, it emits fluorescence that enables clear, real-time visualization of bile ducts using specialized cameras [9-11].

Clinical evidence demonstrates that NIRF imaging has notable safety and efficacy outcomes across multiple surgical applications. Studies reveal biliary structure visualization rates ranging from 71.4% to 100% for the cystic duct (CD), with weighted averages exceeding 96% for key anatomical visualization. Comparative studies show that NIRF identifies biliary anatomy faster than conventional cholangiography. International consensus supports the technology's safety profile, with adverse event rates below 0.003% and no major complications reported in large patient cohorts [12-15]. In this review, we comprehensively highlighted the literature findings regarding the various applications of NIRF imaging in general surgery.

## Review

Fluorescent contrast agents

Fluorescent contrast agents are essential for enhancing visualization during NIRF imaging in general surgery. These agents enable real-time identification of anatomical structures, tumor margins, and vascularization, supporting precision and safety in surgical procedures [16,17].

Classical fluorescent contrast agents, including fluorescein, methylene blue, 5-aminolevulinic acid (5-ALA), and ICG, represent foundational tools in fluorescence-guided surgery. These established agents demonstrate distinct spectral properties and clinical applications. Fluorescein provides visible fluorescence for ophthalmic and neurosurgical applications, methylene blue serves as an NIR sentinel lymph node tracer with 98.5% detection rates when combined with ICG vs. 91.5% alone, 5-ALA accumulates as protoporphyrin IX in tumors, enabling real-time surgical guidance, and ICG remains the gold standard NIR agent for vascular and perfusion imaging with exceptional safety profiles [18-22].

Fluorescent contrast agents work by accumulating in specific tissues or binding to molecular targets. Their fluorescence is then excited by a specific wavelength of light, emitting a signal that can be detected and visualized. The effectiveness of these agents depends on their quantum yield, photostability, and tissue specificity [16,23].

Some agents are designed to activate fluorescence in response to specific microenvironmental conditions, such as acidic pH in tumors. For example, pH-sensitive agents like OBD show increased fluorescence in acidic tumor microenvironments, enhancing tumor margin delineation [24].

Recent progress has enabled the creation of other important targeted agents, such as those conjugated to antibodies or ligands that bind tumor-specific receptors. These agents improve specificity and contrast, allowing for more precise tumor margin detection and identification of metastasis [23-25]. Ideal fluorescent contrast agents exhibit minimal toxicity, rapid clearance, and low nonspecific binding. Water solubility and low phototoxicity are important for clinical safety, especially for repeated or high-dose applications [26-28].

**Figure 1:**
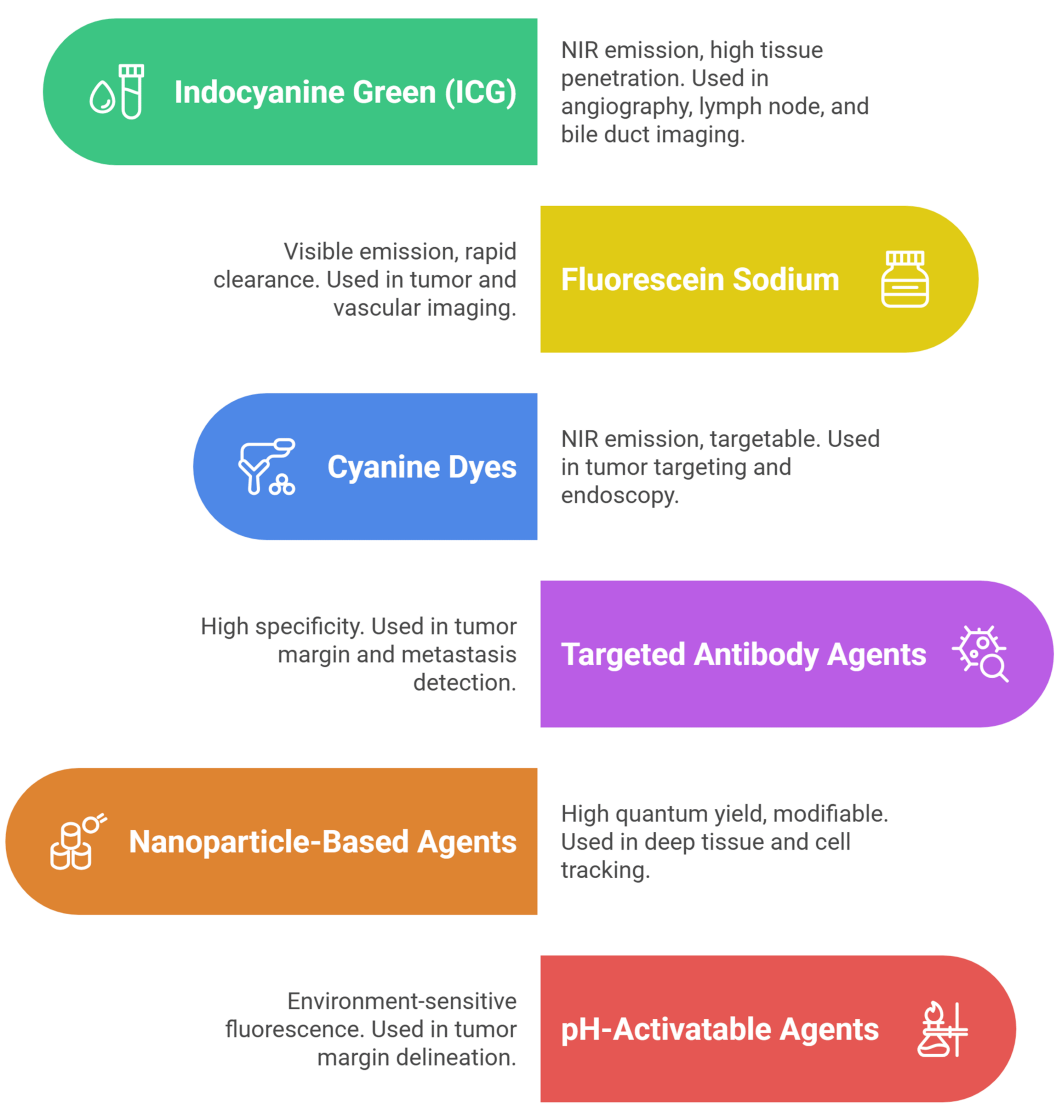
Different fluorescent contrast agents Image credit: This image was created by the author Momen Abdelglil Source: [16-29]

Ongoing research focuses on developing agents with improved targeting, deeper tissue penetration, and multiplexing capabilities for simultaneous imaging of multiple structures. The integration of genetically engineered probes and multicolor imaging is also expanding the potential of NIRF-guided surgery (Figure 1) [24,29].

NIRF imaging in vascularization assessment

**Figure 2:**
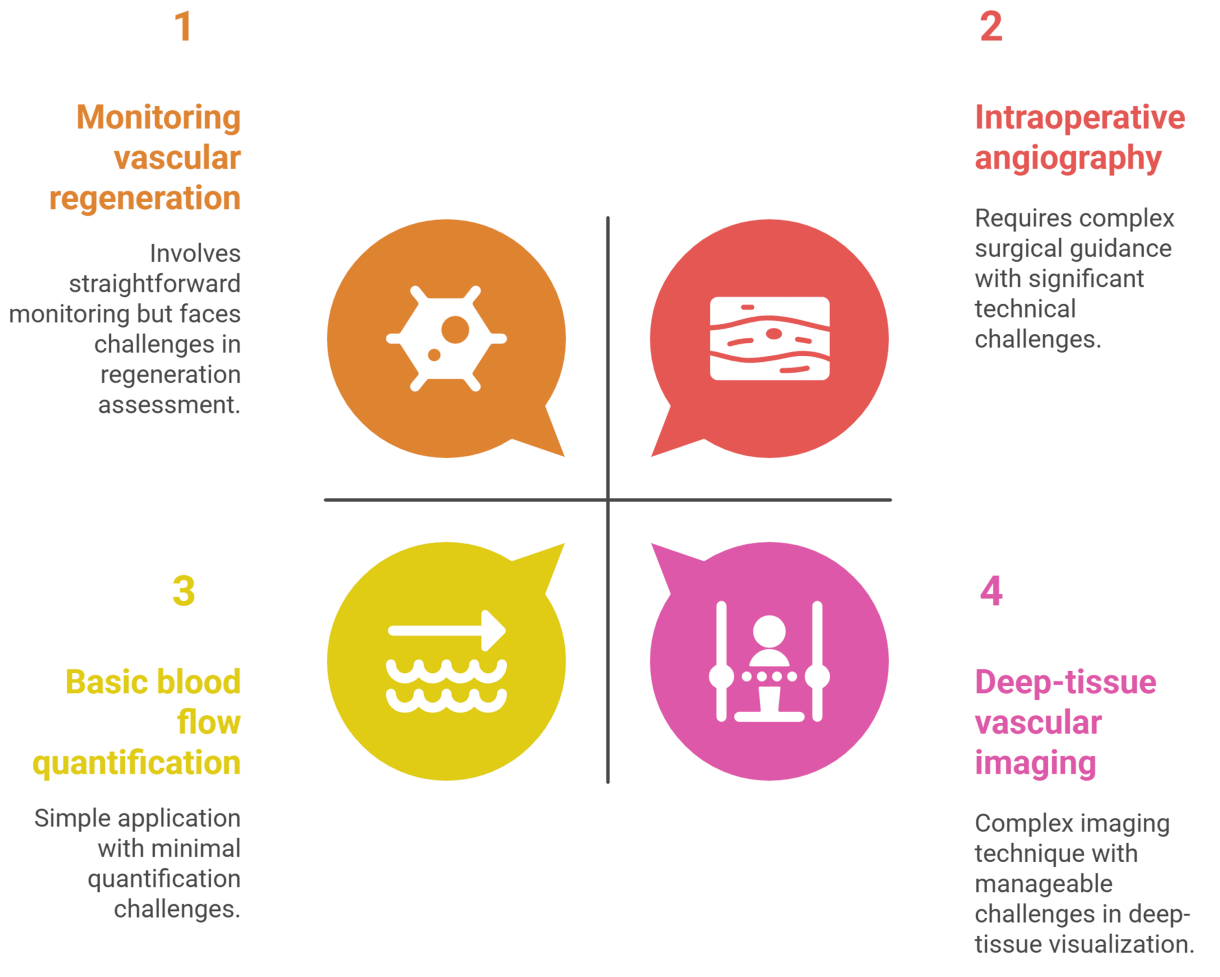
Near-infrared fluorescence imaging in vascularization assessment Image credit: This image was created by the author Momen Abdelglil Source: [34-40]

NIRF imaging, particularly in the second NIR window (NIR-II, 1,000-1,700 nm), has rapidly advanced as a powerful tool for visualizing and quantifying vascular structures and function in vivo. This technology offers significant advantages over traditional imaging modalities, including deeper tissue penetration, higher spatial and temporal resolution, and reduced background autofluorescence, enabling real-time, high-contrast imaging of both macro- and microvasculature [30-33]. NIRF imaging has been applied in a wide range of preclinical and clinical settings, from monitoring vascular regeneration and perfusion in disease models to guiding surgical procedures and assessing vascular pathophysiology (Figure 2) [34-36].

Imaging Performance and Clinical Innovations

The development of novel NIR-II fluorophores, improved imaging systems, and advanced image processing algorithms has further expanded the utility of NIRF imaging for vascular applications [37-39]. NIR-II fluorescence imaging achieves superior spatial (~30 μm) and temporal (<200 ms/frame) resolution for small vessel imaging at depths of 1-3 mm, outperforming traditional NIR-I, micro-CT, and ultrasonography [40].

Innovations in probe design, such as single-walled carbon nanotubes, quantum dots, rare earth-doped nanoparticles, and semiconducting polymer dots, have enabled high-contrast, deep-tissue vascular imaging [31,33,39]. Advanced image processing algorithms, including Hessian matrix enhancement and adaptive optics, further improve vessel visualization and quantification [41-43].

Quantitative Assessment and Functional Imaging

NIRF imaging has been widely used in preclinical models to monitor vascular injury, regeneration, and perfusion, particularly in peripheral arterial disease and ischemia models [37,39,44,45]. Clinically, NIRF imaging with FDA-approved dyes like ICG has been applied for intraoperative angiography, assessment of revascularization outcomes, and detection of atherosclerotic plaques [34-36,46]. NIRF imaging also enables real-time, noninvasive visualization of cerebral, peripheral, and tumor vasculature [32,47,48]. NIRF imaging allows for dynamic quantification of blood flow, vessel density, and perfusion recovery, providing valuable functional information beyond anatomical visualization [39,40].

Despite its advantages, NIRF imaging faces challenges in probe optimization (brightness, biocompatibility, circulation time), standardization of imaging protocols, and quantification accuracy due to blood attenuation and tissue heterogeneity [33,49].

Tumor margin detection

NIRF imaging has emerged as a transformative technology in cancer surgery, providing surgeons with real-time visualization capabilities to identify tumor margins during oncologic procedures. This cutting-edge approach addresses one of the most persistent challenges in cancer surgery: achieving complete tumor removal while preserving healthy tissue. With positive surgical margins occurring in approximately 13% of solid tumor cases, the need for improved intraoperative guidance has become increasingly urgent [50,51].

The significance of complete tumor resection cannot be overstated, as positive margins are associated with a threefold increase in ipsilateral recurrence risk and may account for up to 75% of local recurrences and metastases. NIRF imaging operates within the NIR-I (650-950 nm) and NIR-II (1,000-1,700 nm) spectral windows, where tissue scattering, absorption, and autofluorescence are minimized, enabling superior tissue penetration and contrast [52-54].

Studies have shown that NIR-II imaging can achieve penetration depths ranging from several millimeters to centimeters, with some reports indicating visualization capabilities at tissue depths of up to 23 mm [53].

A groundbreaking advancement in tumor margin detection has emerged through fluorescence lifetime (FLT) imaging in the NIR-II window. Recent studies utilizing folate receptor-targeted ICG nanoprobes have demonstrated superior accuracy in defining tumor margins compared to conventional intensity-based fluorescence imaging. NIR-II FLT imaging achieved 90% accuracy in tumor margin detection, significantly outperforming NIR-II intensity imaging, which achieved only 58% accuracy [51].

EGFR-Targeted Imaging Agents

Epidermal growth factor receptor (EGFR) has become a prominent target for fluorescence-guided surgery, particularly in head and neck squamous cell carcinomas, where EGFR is overexpressed in more than 90% of cases. Two primary EGFR-targeted fluorophores have shown clinical promise [50,55].

Panitumumab-IRDye800

Panitumumab-IRDye800 has emerged as a leading candidate for clinical applications. Recent clinical trials have demonstrated its safety and efficacy in detecting tumor-positive margins with 100% sensitivity while identifying the majority of close margins. In head and neck cancer surgery, panitumumab-IRDye800 changed final resection margins in 21% of cases, potentially preventing the need for adjuvant therapy [55,56].

Cetuximab-IRDye800

Cetuximab-IRDye800 has also shown promising results in clinical studies. With signal-to-background ratios ≥2, this agent enables positive margin detection with 100% sensitivity, 85.9% specificity, and 100% negative predictive value. Studies have demonstrated that fluorescence intensity decreases by factors of 2-3 at 1 mm from the tumor and 4-8 at 5 mm, providing quantitative guidance for margin assessment [25].

Indocyanine Green Applications

ICG, an FDA-approved fluorescent dye, remains the most widely used agent in fluorescence-guided surgery. Recent research has explored ICG applications across various cancer types. A prospective cohort study of 69 breast cancer patients demonstrated that ICG fluorescence detection achieved 81.82% sensitivity and 75.82% specificity for margin assessment. The study revealed dose-dependent effects, with 1 mg/kg dosing providing 87.5% sensitivity compared to 66.67% at 0.5 mg/kg [54].

The Minimally Invasive, Indocyanine-Guided Metastasectomy in Patients with Colorectal Liver Metastases trial investigated ICG-fluorescence-guided minimally invasive liver surgery in eight Dutch centers, demonstrating the potential for real-time resection margin assessment during colorectal liver metastasis surgery [57].

Biliary anatomy visualization

ICG fluorescence cholangiography has proven superior to conventional imaging in identifying critical biliary structures. Studies consistently report enhanced visualization of the CD, common bile duct (CBD), and common hepatic duct [14,58].

Recent clinical studies have demonstrated the significant impact of NIR fluorescence cholangiography in laparoscopic cholecystectomy (LC), particularly in enhancing biliary structure visualization and reducing operative complications. A comprehensive systematic review analyzing over 13,000 patients across 14 studies revealed that ICG fluorescence imaging consistently improved visualization of critical biliary structures compared to conventional white light imaging [14,59].

ICG fluorescence significantly reduces operative time, particularly in complex cases. The weighted average operative time decreased from 96.9 minutes with conventional imaging to 75.3 minutes with ICG fluorescence (p < 0.0001). In challenging anatomical cases, the time reduction was even more pronounced, with studies reporting average reductions of approximately 20 minutes compared to conventional methods [14].

Emergency LC studies have provided additional evidence supporting ICG's efficacy across different severity grades of acute cholecystitis. A prospective cohort study of 81 consecutive patients demonstrated successful visualization of the CD in 56.8% of cases before hepatocystic triangle dissection and 95.1% after complete dissection. Notably, the visualization rates varied significantly between nongangrenous cholecystitis (AAST Grade I) and gangrenous/complicated forms (American Association for the Surgery of Trauma, AAST, Grades II-V), with better outcomes observed in less inflammatory conditions [59].

NIR fluorescence imaging has multiple applications across hepatopancreatobiliary surgery, but two distinct techniques fall under “ICG cholangiography” and should be separated. First, direct intraductal ICG injection (highly diluted ~0.025 mg/mL) provides immediate delineation of the extrahepatic biliary tree and is particularly useful for real-time bile-leak detection during hepatic resection; in a randomized trial of 102 hepatectomies without biliary reconstruction, intraductal ICG cholangiography reduced postoperative bile leaks from 10% to 0% (p = 0.019) [60].

Second, systemic (intravenous) ICG administration exploits hepatobiliary excretion to render the ducts fluorescent without cannulation, aiding anatomical identification (e.g., CBD and CD) during laparoscopic and open procedures; typical regimens use 2.5-10 mg IV given ~30-90-minute preimaging, with clinical series demonstrating reliable duct visualization and favorable signal-to-background over that window [1]. Separately, for anatomical hepatic resection, selective portal-vein ICG injection enables precise segmental and subsegmental staining, and surface mapping to guide parenchymal transection, which has been reported to be successful in the majority of patients undergoing hepatectomy [1,60].

**Figure 3:**
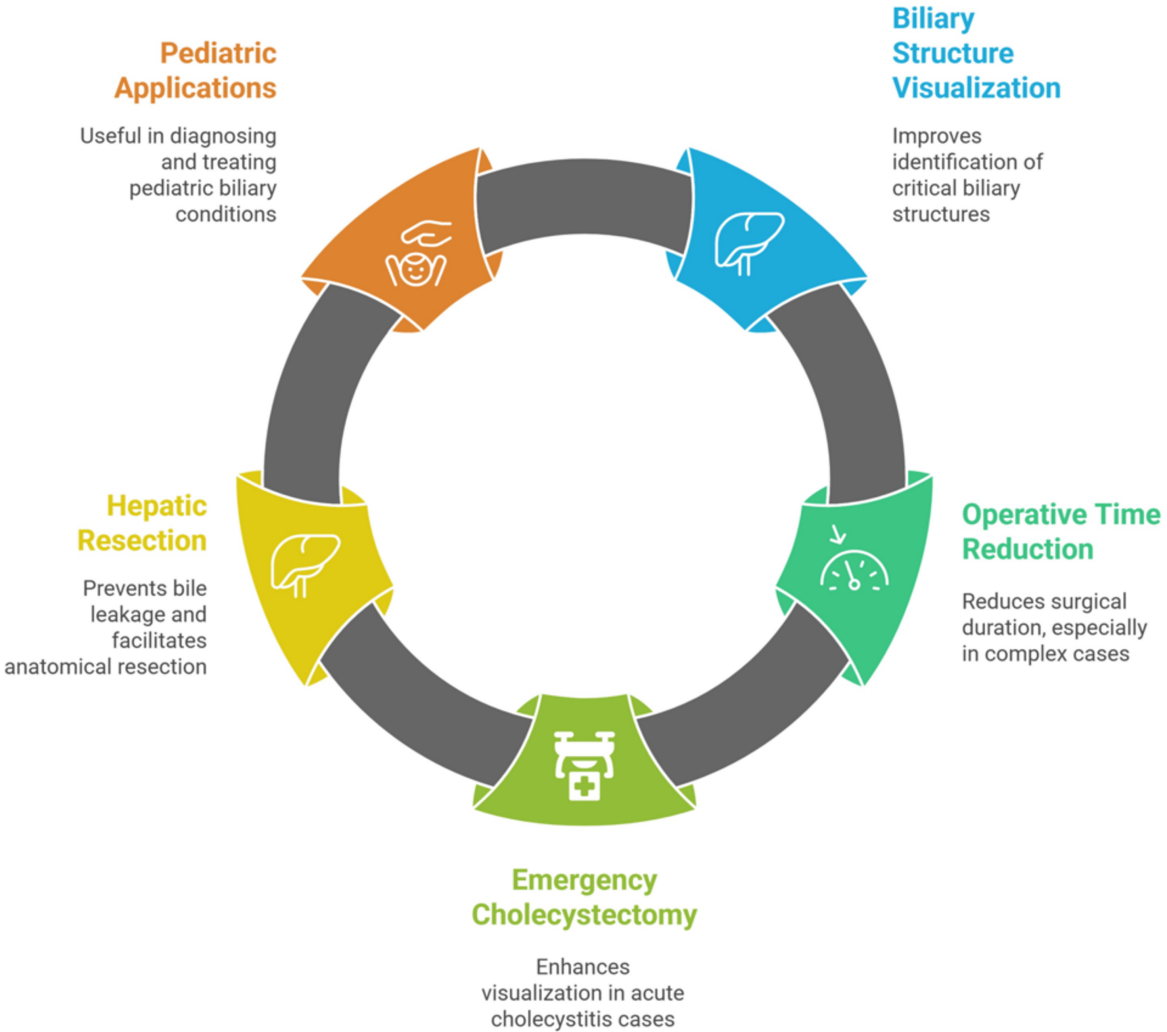
Near-infrared fluorescence imaging in biliary anatomy visualization Image credit: This image was created by the author Momen Abdelglil Source: [14,58-61]

Pediatric applications have shown particular promise, with systematic reviews of 930 pediatric patients demonstrating ICG's utility in biliary atresia diagnosis, choledochal cyst surgery, and liver transplantation. The radiation-free nature of fluorescence imaging makes it especially valuable in pediatric populations, where minimizing radiation exposure is critical (Figure 3) [61].

Future directions and emerging technologies

Beyond ICG, researchers are developing next-generation fluorescent agents specifically designed for biliary imaging. BL-760, a novel NIR fluorophore, demonstrates superior properties, including lower required doses (90 μg/kg), shorter time to biliary excretion, and higher target-to-background ratios compared to ICG. This compound shows promise for rapid biliary-specific excretion and sustained high contrast relative to adjacent liver tissue [62,63].

Second-window NIR imaging (NIR-II, 1,000-1,700 nm) represents another frontier in fluorescence-guided surgery, offering improved tissue penetration and reduced light scattering compared to traditional NIR-I systems. Early clinical trials suggest NIR-II fluorescence imaging may provide enhanced resolution for hepatobiliary applications [64].

Advanced targeted molecular probes are revolutionizing tumor-specific visualization through sophisticated biomarker recognition systems. CD24-targeted NIR-II probes demonstrate exceptional sensitivity and specificity for colorectal neoplasia detection, achieving 92% accuracy for lesions smaller than 1 mm. Biomimetic metallacage nanoparticles with aggregation-induced emission properties enable simultaneous multimodal imaging and immunotherapy guidance. Trop2-targeting peptides labeled with ICG show remarkable potential for breast cancer margin assessment and sentinel lymph node identification [65-67].

Artificial Intelligence Integration

The integration of artificial intelligence (AI) with fluorescence imaging represents a significant advancement in cancer surgery precision. Researchers have developed techniques combining machine learning with short-wave infrared fluorescence imaging, achieving 97.5% per-pixel classification accuracy for tumor identification. This approach utilizes spectral characteristics rather than intensity measurements, providing more robust tumor delineation [68].

AI-driven analysis of FLT imaging has shown promise in automating tumor margin detection and improving diagnostic accuracy. Deep learning models can process complex Fluorescence Lifetime Imaging Microscopy datasets to enhance tumor classification and therapy response assessment [69].

Robotic and Advanced Surgical Applications

Integration of fluorescence imaging with robotic surgical systems has expanded the technology's applications. The da Vinci Firefly® system enables seamless switching between white light and fluorescence modes without disrupting surgical workflow. Robotic hepatobiliary procedures utilizing fluorescence guidance have demonstrated successful identification of biliary structures in 99% of cases, with 83% detecting all four key anatomic ducts [70].

Advanced applications include simultaneous fluorescence cholangiography and angiography, enabling identification of both biliary structures and the cystic artery through sequential ICG administration. This dual-imaging approach has shown 89.3% success rates for cystic artery identification, potentially improving surgical safety in complex dissections [71].

## Conclusions

NIRF imaging has revolutionized general surgery by offering real-time, high-contrast views of blood vessels, tumor margins, and biliary anatomy. ICG remains the standard agent, proving safe and effective across procedures. Advances in NIR-II fluorophores, activatable probes, and targeted dyes (EGFR, folate receptor) have enhanced depth, specificity, and margin detection, while integrating fluorescence guidance with robotic systems and AI analysis has streamlined workflows and boosted surgical accuracy.

Emerging NIRF technologies promise even greater precision. Novel agents such as BL-760 and CD24-targeted NIR-II probes are in clinical trials, delivering superior target-to-background ratios at reduced doses. Multiplexed imaging, FLT analysis, nanoparticle-based, and genetically encoded fluorophores aim to refine tumor delineation and track cellular processes intraoperatively. Coupled with AI-driven margin assessment and predictive modeling, these innovations will further personalize and inform surgical decision-making for improved patient outcomes.
